# Diet Quality and Nutrient Intake of Urban Overweight and Obese Primarily African American Older Adults with Osteoarthritis

**DOI:** 10.3390/nu10040485

**Published:** 2018-04-13

**Authors:** Sevasti Vergis, Linda Schiffer, Tiffany White, Andrew McLeod, Neda Khudeira, Andrew Demott, Marian Fitzgibbon, Susan Hughes, Lisa Tussing-Humphreys

**Affiliations:** 1 Department of Medicine, 1747 W. Roosevelt Road, Chicago, IL 60608, USA; svergi3@uic.edu (S.V.); nedakhudeira@gmail.com (N.K.); 2 Department of Kinesiology and Nutrition, University of Illinois at Chicago, 1919 W. Taylor Street, Chicago, IL 60612, USA, white35@uic.edu (T.W.); amcleo2@uic.edu (A.M.); 3 Honors College, University of Illinois at Chicago, 828 S. Halsted Street, Chicago, IL 60607, USA; 4 Institute for Health Research and Policy, 1747 W. Roosevelt Road, Chicago, IL 60608, USA; lschiff@uic.edu (L.S.); mlf@uic.edu (M.F.); 5 Center for Research on Health and Aging, Institute for Health Research and Policy, University of Illinois at Chicago, 1747 W. Roosevelt Road, Chicago, IL 60608, USA; ademot1@uic.edu (A.D.); shughes@uic.edu (S.H.); 6 Department of Pediatrics, University of Illinois at Chicago, 1853 W. Polk Street, Chicago, IL 60612, USA; 7 University of Illinois Cancer Center, University of Illinois at Chicago, 818 S. Wolcott Avenue, Chicago, IL 60612, USA; 8 Department of Community Health Sciences, School of Public Health, University of Illinois at Chicago, 1603 W. Taylor St, Chicago, IL 60612, USA

**Keywords:** diet quality, nutrient intake, African Americans, older adults, osteoarthritis

## Abstract

Diet quality may be a unique target for preventing and managing obesity-related osteoarthritis (OA). Using the Healthy Eating Index-2010 (HEI-2010), this study examined the nutrient intake and diet quality of 400 urban overweight and obese primarily African American older adults with self-reported lower extremity OA. Associations between sociodemographic and health-related factors and diet quality were explored. Participants (mean age 67.8 years, SD 5.9) were included. Habitual dietary intake was assessed using a food frequency questionnaire (FFQ). Nutrient intake and diet quality were calculated from the FFQ. Results indicated that diet quality needs improvement (HEI-2010: 66.3 (SD 10.5)). Age, body mass index, employment (multivariable model only), and OA severity (bivariate model only) were significant predictors of HEI-2010 total score in linear models. Mean intakes for fiber, calcium, and vitamin D were below recommendations, while percentage of calories as total fat exceeded recommendations. These findings can inform future dietary intervention trials and public health messaging for a sub-population at a high risk for obesity-related OA.

## 1. Introduction

The population of older adults in the United States (U.S.), specifically adults over the age of 65 years, rose dramatically from 35 million in 2000 to 49.2 million in 2016 [1]. This shift in U.S. demographics comes with an increase in the incidence and prevalence of chronic diseases that place a significant toll on an individual’s quality of life as they age as well as on the U.S. healthcare system [2]. Osteoarthritis (OA) is one such condition affecting over 30 million older adults in the U.S., with African Americans disproportionately burdened by large joint (i.e., kip, knee) OA [3,4]. This health inequity is largely attributed to higher rates of overweight and obesity among older African American adults [5], particularly African American women, given excess body weight is a significant modifiable risk factor for OA [6].

There are currently two major causal theories explaining the association between overweight/obesity and OA. The mechanical theory focuses on the effects of loading and cartilage degradation—specifically, the repetitive application of a higher load on large joints (i.e., knee and hip) that leads to the degeneration of articular cartilage and sclerosis of the subchondral bone [7]. The second theory, known as the metabolic theory, surmises that OA arises through an indirect action of elevated pro-inflammatory cytokines stemming from increased adipose tissue that leads to joint degradation or, along with the mechanical stress, exacerbates joint degeneration [8]. Thus, interventions aimed at reducing excess body weight and systemic inflammation may have a significant role in OA prevention, progression, and symptom management.

Diet is a common intervention target to reduce excess body weight and systemic inflammation, and has been the focus of several studies in older overweight and obese adults with OA. The majority of studies have focused on calorie restriction to promote weight loss, demonstrating promising short-term effects for weight loss, systemic inflammation, and physical function [9,10]. However, difficulties maintaining weight loss in the long term [11] and the untoward effects of calorie restriction on lean muscle mass [12] has led researchers and clinicians to seek alternative dietary approaches that may be more maintainable but comparable in regard to body fat and inflammatory results [13].

Diet quality is a relatively new concept in nutritional epidemiology focused on the quality and synergy of whole foods included in a diet pattern versus single nutrients [14]. Diet quality can be determined using data-driven approaches such as principal components analysis as well as a priori methods that assess adherence to existing population-based dietary recommendations (e.g., United States Department of Agriculture’s (USDA) Dietary Guidelines for Americans (DGAs)). One common a priori method for determining diet quality is the Healthy Eating Index (HEI) [15]. HEI assesses adherence to a given set of DGAs; higher scores reflect greater adherence [16]. Operationalizing and quantifying diet quality allows one to examine associations with health status [14]. In recent studies, improving diet quality was associated with less weight gain over time [17], greater weight loss [18], and reduced systemic inflammation independent of weight loss [19]. Moreover, superior diet quality is associated with lower OA prevalence [20] and reduced risk for symptomatic knee OA in women [13]. Together, these findings suggest that improving diet quality may be a key target for preventing and managing obesity-related OA. 

In order to design effective interventions that target diet quality, an understanding of current diet quality is essential. To date, no studies have examined diet quality in older overweight and obese African American adults with OA—a subpopulation at a disproportionately burdened by OA [3,4]. However, we do know from general population-based studies that older African American adults have lower diet quality based on HEI compared to other racial/ethnic groups [21]. The primary aim of this study was to describe the diet quality (HEI-2010) and nutrient intake of urban overweight and obese primarily African American older adults with self-reported lower extremity OA. The secondary aim was to examine associations between sociodemographic and health-related factors and diet quality. Such data will help to guide the development of tailored lifestyle interventions to promote healthy aging in populations at the greatest risk for OA [22].

## 2. Materials and Methods

### 2.1. Study Design and Setting

This study is a cross-sectional analysis of data collected at baseline from the Fit & Strong! Plus comparative effectiveness trial (R01AG039374; NCT03180008) that was designed to compare the impact of physical activity versus physical activity plus dietary weight management on body weight, diet quality, physical activity, and OA symptoms in overweight and obese older adults with self-reported lower extremity OA. A detailed description of the trial’s design and methods has been published elsewhere [23]. Briefly, subjects were recruited through advertisements and presentations at various community facilities in the city of Chicago including Chicago Park District sites and City of Chicago senior centers; emails were also sent from the Arthritis Foundation to listserv members residing in Chicago. The study was reviewed and approved by the University of Illinois at Chicago Institutional Review Board. All subjects provided written informed consent prior to study participation.

### 2.2. Study Population and Eligibility Criteria

Interested individuals were screened for eligibility over the phone. Eligibility criteria included: self-reported diagnosis of lower extremity OA described as pain in or around at least one knee or hip most days in the past month, or symptoms of pain and/or stiffness in or around hips, knees, ankles, feet, or lower back on most days of at least one month in the past six months; no current participation in a structured physical activity or weight management program and engaging in less than 150 min of moderate to vigorous physical activity weekly; self-reported height and weight consistent with a BMI (body mass index) of 25–50 kg/m^2^ (confirmed at the baseline visit); aged 60 years or older; and ability to participate in study procedures, measurements, and interventions. Exclusion criteria included a score of 3 or more on the nine-item Mini Mental Status Questionnaire [24]; uncomplicated hip or knee surgery within the past 6 months; surgery with complications within the past year; plans for hip or knee surgery within the next year; steroid injections in either hip or knee in the past three months; diagnosis of rheumatoid arthritis, uncontrolled diabetes, or other health conditions that might interfere with exercise. The Exercise and Screening for You (EASY) screener was also administered to subjects to identify health reasons that may contraindicate participation in physical activity programs. Individuals who identified one or more high-risk condition on the EASY screener were required to obtain approval from their physician before participating. Four hundred and thirteen individuals completed baseline assessments and were enrolled in the trial. 

### 2.3. Study Measures

Habitual dietary intake: All study subjects completed the semi-quantitative 110-item Block 2005 Food Frequency Questionnaire (FFQ) at baseline. The FFQ was administered by trained research staff and facilitated using a standardized portion guide. The FFQ was designed to assess habitual dietary intake of foods over the past year and current use of a variety of common dietary supplements (e.g., multivitamins, calcium, iron). Nutrient values that were calculated from the FFQ included: mean energy intake and percent of energy intake from fat, mean intake of fiber per 1000 calories, and mean intake of total fat, saturated fat, monounsaturated fat, polyunsaturated fat, trans fat, carbohydrates, protein, total fiber, sugar, cholesterol, sodium, calcium, vitamin D, vitamin C, iron, and vitamin B12. NutritionQuest (Berkeley, CA, USA) processed the FFQ survey booklets using a proprietary nutrient database developed from the USDA Food and Nutrient Database for Dietary Studies (FNDDS), version 1.0 [25] to obtain mean daily intake of macro- and micronutrients and HEI-2010 total and component scores. Only plausible (i.e., consuming 500–5000 calories daily) [26] and valid (i.e., <10 missing responses) [27] surveys were used for the analysis (*n* = 13 were excluded). 

HEI-2010 is a commonly used measure of diet quality that assesses adherence to the 2010 DGAs [28]. HEI-2010 consists of 12 diet components that encourage adequacy (nine components) and moderation (three components) of foods and nutrients summing from 0–100 points. The nine adequacy components are total fruit, whole fruit, total vegetables, greens and beans, whole grains, dairy, total protein foods, seafood and plant proteins, and fatty acids. The three moderation components are refined grains, sodium, and empty calories (calories from solid fats, alcoholic beverages, and added sugars). Higher scores in all components indicate closer conformance to the DGAs. Thus, higher intakes in the adequacy components receive higher scores and higher intakes in the moderation components receive lower scores. HEI-2010 uses an energy density approach to scoring (i.e., per 1000 calories or as a percent of calories), making it unnecessary to control for mean energy intake during statistical analysis [28].

Sociodemographic characteristics: A sociodemographic survey was administered to examine subjects’ self-reported gender, age, race/ethnicity, educational attainment, relationship status, household income, health insurance, and current employment status.

Anthropometric measures: Body weight was measured twice using a digital scale (Tanita, Arlington Heights, IL, USA). Height was measured twice using a portable stadiometer (seca, Birmingham, UK). BMI was calculated from weight (kg) and height (m^2^) and was used to classify subjects into the categories of overweight (25–29.9 kg/m^2^), obesity class I (30–34.9 kg/m^2^), obesity class II (35–39.9 kg/m^2^), or obesity class III (>40 kg/m^2^). 

Osteoarthritis severity: The Western Ontario McMaster Universities Osteoarthritis Index (WOMAC) was used to evaluate symptoms of lower extremity OA in the study subjects. The WOMAC evaluates symptoms of pain (five items; score range 0–20), stiffness (two items; score range 0–8), and physical function (17 items; score range 0–68) for a total score of 0–96. A higher score reflects greater difficulties related to OA [29]. The validity of the WOMAC in patients with hip or knee OA has been demonstrated [29].

### 2.4. Statistical Analysis

Of the 413 participants in the trial, 13 had diet records with implausible estimates of energy intake (<500 or >5000 calories). These participants were excluded from all analyses described here (*n* = 400). We used *t*-tests with pooled variance to test for differences in the HEI total score by participant demographics, BMI category (overweight/obese), and WOMAC global score (dichotomized at the median for the sample). We explored potential predictors of HEI scores using linear models with the HEI total score or a component score as the dependent variable and demographic variables, BMI, and the WOMAC global score as covariates. In these models, BMI and the WOMAC global score were entered as continuous variables. All analyses were conducted using SAS v 9.4.

## 3. Results

Four hundred and thirteen participants were recruited, and 400 had what were deemed plausible and valid FFQs and were included in the analysis. The sociodemographic and health characteristics of the participants are reported in Table 1. The majority of the sample was female and African American. The mean age of the participants was 67.8 years (SD = 5.9). Eighty percent of all subjects reported having completed some college or technical school or having a college degree; the median household income was $25,000. Just over a quarter reported being married or a member of an unmarried couple. 

The mean BMI at baseline was 34.8 kg/m^2^ (SD = 5.5), with 45% classified as obesity class II and III. Based on the WOMAC, subjects reported having a moderate amount of OA-related symptoms. 

The habitual nutrient intake of the participants by gender along with recommended intake targets is reported in Table 2. The mean energy intake for men and women was 1693 kcals (SD = 702) and 1560 kcals (SD = 711), respectively. The percent of energy consumed as total fat was similar for men and women at 39.8% and 39.9%, respectively, exceeding daily recommendations. The mean intake of dietary fiber intake per 1000 calories was 8.8 g and 10.1 g for men and women, respectively. Mean values for sodium were above and for calcium and vitamin D were below daily recommendations for both men and women [30,31,32].

Table 3 presents subjects’ HEI-2010 mean total and component scores by gender and categorizes their diet quality into three categories: “poor” (score range 0–50), “needs improvement” (score range 51–80), and “good” (score range 81–100) [33]. The overall diet quality needed improvement for both men and women, as indicated by the mean total scores of 64.3 and 66.6, respectively. More men (12.3%) compared to women (7.3%) had “poor” diet quality. Of the adequacy components, whole grains and dairy were particularly low, with mean scores of 4.4 (both genders) and 4.0 and 3.9 out of 10 for men and women, respectively. The remaining adequacy components received higher scores; notably, total protein foods had the greatest scores of 4.9 and 4.7 out of 5 for men and women, respectively. Of the three moderation components, refined grains received scores of 9.1 and 9.0 out of 10 for men and women, respectively, indicating relative compliance with recommendations, and for empty calories the scores were 11.4 and 12.4 out of 20 for men and women, respectively, indicating only modest compliance. The final moderation component, sodium, received scores of 4.8 and 4.4 out of 10 for men and women, respectively, indicating poor compliance with this recommendation. The men had notably lower component scores for total vegetables, greens and beans, and empty calories compared to the women. 

There was no significant difference in the HEI-2010 total score based on sex, race, education, relationship status, or employment (Table 4). However, significant differences were observed based on age, BMI, and WOMAC global score. Specifically, a lower HEI-2010 total score was reported for subjects less than 70 years old compared to those ≥70 years old (65.4 vs. 68.4; *p* = 0.009), obese compared to overweight (65.7 vs. 68.8; *p* = 0.02), and for those with a WOMAC global score greater than or equal to the median compared to those with a global score less than the median (65.2 vs. 67.4; *p* = 0.04). 

The sociodemographic and health-related characteristics were tested as possible predictors of HEI-2010 total as well as HEI-2010 component scores in multivariable linear regression models (Table 5). Age (β = 0.21 (SE 0.09)), employment (β = 3.26 (SE 1.56)), and BMI (β = −0.20 (SE 0.10)) were independent predictors of the HEI-2010 total score when adjusting for the other sociodemographic and health-related variables. When examining the HEI-2010 component scores, male gender was a significant inverse predictor of total vegetable score (β = −0.70 (SE 0.17)), and, greens and beans score (β = −0.53 (SE 0.17)), and a positive predictor of total protein score (β = 0.22 (SE 0.09)) when adjusting for the other sociodemographic and health-related variables. Age was a significant positive predictor of total fruit score (β = 0.03 (SE 0.01)) and whole fruit score (β = 0.03 (SE 0.01)). College graduate education level was a positive predictor of total fruit score (β = 0.34 (SE 0.15)) and surprisingly a negative predictor of whole grain score (β = −0.76 (SE 0.30)). Race other than African American was a significant positive predictor of whole grain score (β = 1.17 (SE 0.53)) and dairy score (β = 1.49 (SE 0.42)) as well as a negative predictor of refined grain score (β = −1.22 (SE 0.28)). WOMAC global score was a positive predictor of dairy score (β = 0.02 (SE 0.01)) and a negative predictor of fatty acids score (β = −0.02 (SE 0.01)). Finally, full or part-time employment was a positive predictor of empty calories score (β = 1.41 (SE 0.69)).

## 4. Discussion

Although weight loss has been shown to have a promising effect on OA progression, functional outcomes, and symptom management [9,10], weight loss is difficult to achieve and maintain for most individuals, including African Americans [39]. Diet quality may be a more salient intervention target for managing obesity-related OA, given that it involves changing one’s dietary pattern but not restricting calories. However, there is limited research examining diet quality and nutrient intake of overweight and obese African American older adults with OA, despite the fact that this population is disproportionately burdened by both chronic conditions [3,4,5]. From a public health and clinical perspective, information on diet quality is crucial to inform and target efforts addressing the nutritional needs and improvement of dietary intakes of this at-risk segment of the U.S. population [40]. To advance the literature, we conducted a cross-sectional analysis to describe the diet quality (HEI-2010) and nutrient intake of 400 urban overweight and obese primarily African American older adults with self-reported lower extremity OA.

Overall, we report that the diet quality of both men and women in our cohort needs improvement [33]. Our findings are consistent with the mean HEI-2010 total score reported for a representative sample of U.S. adults aged 65 years and older (69.3 (SD = 1.8)) [41]. While no studies have examined associations between diet quality measured by HEI and OA risk, progression, or symptom management, one study reported that higher mean HEI-2005 total score was associated with better gait speed in U.S. older adults, a marker of physical function often compromised in those with OA [42]. Two studies examined associations between diet quality, defined as adherence to a Mediterranean diet [20] and dietary glycemic index [13], and OA-related outcomes. Higher adherence to a Mediterranean diet, a largely plant-based eating pattern including olive oil as the predominant fat, was associated with lower OA prevalence in the large North American OA Initiative cohort [20]. In another study of Korean adults, adherence to a diet with a low glycemic index, indicative of low consumption of refined carbohydrates, was associated with decreased risk of symptomatic knee OA in women [13]. The authors of both studies surmised that the positive OA outcomes were attributed to the anti-inflammatory [43,44], anti-oxidative [45,46], and cartilage preserving [47,48,49] effects of the higher quality diets. However, randomized controlled trials are needed to confirm these findings and to understand the underlying biological mechanisms linking diet quality and OA. 

In our cohort, empty calories, whole grains, and dairy were among the lowest scoring of the 12 HEI-2010 components. Compared to HEI-2010 component scores reported for the general population of older U.S. adults (≥65 years), our mean whole grains score was similar (4.4 (SD 2.9) vs. 4.23 (SD 0.34)), while empty calories (12.2 (SD 4.7) vs. 14.99 (SD 0.44)) and dairy scores (3.9 (SD 2.3) vs. 5.99 (SD 0.16)) were lower [41]. A lower dairy score was somewhat expected given the higher rates of perceived and actual lactose intolerance in African Americans [50]. Moreover, aligning with the same diet quality components, we also observed that mean nutrient intakes for fiber, calcium, and vitamin D were below recommendations, while the percentage of calories as total fat exceeded recommendations. Intake of empty calories, whole grains/fiber, and dairy may be relevant to OA. Specifically, “empty calories” from solid fats, added sugars, and alcohol [28] may contribute to unhealthy weight gain [51], increased production of pro-inflammatory cytokines and adipokines, and oxidative stress [45,52,53], all of which may play an important role in the mechanical and metabolic pathogenesis of OA [7,8]. Similarly, low intake of whole grains, which are rich in fiber and antioxidant nutrients, is associated with poor body weight regulation and increased systemic inflammation [54,55]. The association between dairy foods and OA is somewhat muddled. In a large cohort study of Australian adults, greater dairy consumption was associated with increased risk for hip replacement in men with hip OA [56]. Meanwhile, in a large North American cohort, higher milk consumption was associated with reduced OA progression in women [57]. Some argue that it is not dairy foods per se that are protective for OA but the nutrients they contain such as calcium, vitamin D, and B vitamins, given their role in bone and muscle health [58,59,60]. Clearly, randomized dietary intervention trials are needed to corroborate observational findings and to understand the relationships between empty calories, whole grains/fiber, dairy, and OA. Given the observed inadequacy, these components of diet quality may be important intervention targets for older African American adults with OA. 

We evaluated associations between diet quality and sociodemographic and health characteristics of the study participants. In the bivariate analyses, we found better diet quality in adults over 70 years old compared to those aged 60–69 years, in overweight subjects compared to obese, and in those with lower global scores on WOMAC compared to those with higher global scores. A study by Reedy et al. [61] of diet quality and all-cause, cardiovascular disease, and cancer mortality among older adults found that younger age and higher BMI were both significant inverse predictors of HEI-2010 total scores. In other studies examining predictors of diet quality in U.S. adults, female gender, non-Hispanic white race, and higher perceived social support among men were significant predictors of superior diet quality based on HEI, while living alone was associated with lower diet quality [40,62,63]. To our knowledge, existing studies have not evaluated associations between HEI-2010 and WOMAC global scores. However, in the study by Veronese and colleagues, greater adherence to a Mediterranean diet was associated with lower WOMAC global scores among adults with OA [20], suggesting that OA severity (i.e., pain, stiffness, and overall physical function) is linked with diet quality. However, confirmation in randomized controlled trials is needed. 

We also examined associations between the HEI-2010 component scores and sociodemographic and health characteristics of the participants. Older age was associated with higher total fruit and whole fruit scores; male gender was associated with lower total vegetables and greens and beans scores, as well as higher total protein score; self-identifying race as other than African American was associated with higher whole grains and dairy scores and lower refined grains score; full or part-time employment was associated with higher empty calories score; and a higher WOMAC global score was associated with lower fatty acid score and higher dairy score. Partially corroborating our findings, in a representative sample of U.S. adults, older age was associated with higher intake of total and whole fruits; male gender was associated with lower intake of vegetables including greens and beans; and identifying as non-Hispanic black was associated with lower whole grain intake [64,65,66]. Although no studies have examined associations between WOMAC global score and HEI components, higher intake of saturated vs. unsaturated fatty acids is associated with greater systemic inflammation and progression of lower extremity OA [67], providing some biological plausibility to our observed association. 

A major strength of our study was the assessment of diet quality using HEI-2010 and the examination of sociodemographic and health predictors of diet quality in a majority urban African American sample of overweight and obese older adults with lower extremity OA. Dietary behaviors of racial/ethnic minority older adults have been largely understudied despite disparities in several diet-related chronic diseases and the importance of this data to designing appropriate interventions. However, this study is not without limitations. First, we acknowledge that there may be many other factors that contribute significantly to diet quality and nutrient intake of older adults that were not addressed, particularly concerning racial/ethnic minorities living in urban areas, including food-access, food security, the physical environment, living conditions, real and perceived discrimination, social support, and cohesion [40,63,68,69]. Future studies should examine how these factors relate to diet quality in this subgroup. Second, an FFQ is biased by poor participant recall of dietary intake and by participant’s provision of socially desirable responses [66]. Further, underreporting of food intake has been observed in obese individuals as well as African American women [70,71,72]. Our sample size may have limited our ability to effectively examine diet patterns for subgroups (e.g., men vs. women). Because the analysis was cross-sectional, we were also not able to infer causal associations between diet quality and the sociodemographic and health factors that we examined. Lastly, it is important to acknowledge that generalizability of our findings may be limited given that the individuals that we assessed were enrolling in a lifestyle intervention program and thus may not be representative of the wider population of urban overweight and obese older African Americans adults with lower extremity OA.

## 5. Conclusions

Diet quality and components of diet quality including empty calories, whole grains/fiber, and dairy may play an important role in OA risk, progression, and symptom management. Still, well-designed randomized dietary intervention trials are needed to provide conclusive evidence for the role of diet quality in OA-related outcomes. In the current study of urban, primarily African American overweight and obese older adults with lower extremity OA, overall diet quality (HEI-2010) and component scores for empty calories, whole grains, and dairy needed improvement, and intakes of several nutrients including vitamin D, calcium, and fiber did not meet sex- and age-specific recommendations. Our findings can be used to inform future dietary intervention trials and public health messaging for this at-risk group. 

## Figures and Tables

**Table 1:** Characteristics of urban overweight and obese primarily African American older adults with osteoarthritis (*n* = 400).

	Mean or %	SD or *n*
Age, years	67.8	5.9
60–69	70%	279
≥70	30%	121
Gender		
Female	86%	343
Male	14%	57
Race		
Black or African-American, not Hispanic	92%	367
White, not Hispanic	4%	17
Hispanic	1%	5
Multiracial/other	3%	11
Education, years	14.2	1.9
Not HS graduate, *n* (%)	6%	22
HS graduate/GED	15%	61
Some college or technical school	43%	170
College graduate	37%	147
Employed full or part-time	13%	53
Relationship status		
Married or member of unmarried couple	26%	104
Divorced or separated	36%	145
Widowed	22%	88
Never married	16%	63
Income, median (*n* = 338)	25,000	
Health insurance ^a^		
Medicare	68%	271
Medicaid	17%	68
Private/supplemental	42%	166
WOMAC global score (0–96) ^b^	26.7	17.2
Pain subscale (0–20)	5.6	4.0
Stiffness subscale (0–8)	3.2	1.7
Physical functioning subscale (0–68)	17.9	12.7
BMI, kg/m^2^	34.8	5.5
BMI category		
Overweight (25–29.9 kg/m^2^)	20%	78
Obesity class I (30–34.9 kg/m^2^)	36%	144
Obesity class II (35–39.9 kg/m^2^)	25%	100
Obesity class III (≥40 kg/m^2^)	20%	78

^a^ Percentage of participants reporting each type of insurance; some participants reported more than one type of insurance; ^b^ A higher score indicates greater difficulties due to osteoarthritis (OA). Abbreviations: BMI, body mass index; GED, General Educational Development; HS, high school; WOMAC, Western Ontario McMaster Universities Osteoarthritis Index.

**Table 2:** Habitual nutrient intake of urban overweight and obese primarily African American older adults with osteoarthritis (*n* = 400).

Variable	Intake				Recommendation			
	Men (*n* = 57)		Women (*n* = 343)		Men		Women	
	Mean	SD	Mean	SD	51–70 Years	>70 Years	51–70 Years	>70 Years
Energy, kcal [34]	1693	702	1560	711	2000–2200	2000	1600	1600
Total fat, g [34]	75.1	34.1	70.0	35.6	Not determined	Not determined	Not determined	Not determined
Fat, % calories [34]	39.8	5.9	39.9	7.1	20–35	20–35	20–35	20–35
Carbohydrates, g [34]	189.7	86.6	178.5	82.0	130	130	130	130
Protein, g [34]	65.0	30.5	59.4	31.8	56	56	46	46
Fiber, g [34]	14.2	6.2	15.3	7.9	30	30	21	21
Fiber, g/1000 kcal	8.8	3.0	10.1	3.7	14	14	14	14
Saturated fat, g [32]	22.1	11.8	19.9	10.7	<10% of intake	<10% of intake	<10% of intake	<10% of intake
Monounsaturated fat, g [35]	30.4	14.2	28.0	14.3	Majority of fats	Majority of fats	Majority of fats	Majority of fats
Polyunsaturated fat, g [36]	16.8	7.0	16.7	9.0	Majority of fats	Majority of fats	Majority of fats	Majority of fats
Trans fats, g [32]	2.3	1.6	2.0	1.2	As low as possible	As low as possible	As low as possible	As low as possible
Sugars, g [32]	94.9	55.1	88.9	48.6	<10% of energy	<10% of energy	<10% of energy	<10% of energy
Cholesterol, mg [34]	282.2	193.5	227.7	147.0	As low as possible	As low as possible	As low as possible	As low as possible
Sodium, mg [32]	2673	1212	2524	1274	<2300	<2300	<2300	<2300
Calcium, mg [30]	632.1	386.0	586.3	296.8	1000	1200	1200	1200
Vitamin D, IU [30]	120.7	84.8	90.7	76.1	600	800	600	800
Vitamin C, mg [37]	94.0	67.1	100.6	62.8	90	90	75	75
Iron, mg [31]	11.7	4.7	10.9	5.5	8	8	8	8
Vitamin B12, mcg [38]	4.7	2.8	3.8	2.8	2.4	2.4	2.4	2.4

Abbreviation: SD, standard deviation.

**Table 3:** Healthy Eating Index-2010 (HEI-2010) total and component scores of urban overweight and obese primarily African American older adults with osteoarthritis (*n* = 400).

	Men (*n* = 57)		Women (*n* = 343)	
	Mean or %	SD or *N*	Mean or %	SD or *N*
Healthy Eating Index-2010 (0–100)	64.3	10.7	66.6	10.4
Total fruit (0–5)	3.5	1.5	3.7	1.4
Whole fruit (0–5)	3.6	1.5	3.9	1.4
Total vegetables (0–5)	3.4	1.3	4.1	1.1
Greens and beans (0–5)	3.8	1.5	4.3	1.3
Whole grains (0–10)	4.4	3.0	4.4	2.9
Dairy (0–10)	4.0	2.1	3.9	2.3
Total protein foods (0–5)	4.9	0.4	4.7	0.7
Seafood and plant protein (0–5)	4.1	1.3	4.2	1.2
Fatty acids (0–10)	7.3	2.5	7.5	2.4
Refined grains (0–10)	9.1	1.3	9.0	1.6
Sodium (0–10)	4.8	2.8	4.4	3.0
Empty calories (0–20)	11.4	5.3	12.4	4.5
HEI-2010 category				
Poor (0–50)	12.3%	7	7.3%	25
Needs improvement (51–80)	82.5%	47	86.0%	295
Good (81–100)	5.3%	3	6.7%	23

Abbreviation: SD, standard deviation.

**Table 4:** Healthy Eating Index-2010 total score by subject characteristics.

	*n*	Mean	SD	*p* ^a^
Sex				0.13
Female	343	66.6	10.4	
Male	57	64.3	10.7	
Age, years				0.009
60–69	279	65.4	10.3	
≥70	121	68.4	10.6	
Race				0.55
African-American	367	66.2	10.4	
Other	33	67.4	11.6	
Education				0.15
College graduate	147	67.3	10.7	
Not college graduate	253	65.7	10.3	
Relationship status				0.80
Married or unmarried couple	104	66.1	10.5	
Other	296	66.4	10.5	
Employment				0.07
Full or part-time	53	68.7	10.2	
Not employed	347	65.9	10.5	
BMI, kg/m^2^				0.02
Overweight (25–29.9)	78	68.8	9.2	
Obese (≥30)	322	65.7	10.7	
WOMAC global score				0.04
<24 (median)	199	67.4	10.5	
≥24	201	65.2	10.4	

^a^ From *t*-tests with pooled variance. Abbreviations: BMI, body mass index; WOMAC, Western Ontario McMaster Universities Osteoarthritis Index.

**Table 5:** Predictors of Healthy Eating Index-2010 (HEI-2010) total and component scores in urban overweight and obese primarily African American older adults with osteoarthritis.

	HEI-2010 (R^2^ = 0.06)			Total Fruit (R^2^ = 0.06)			Whole Fruit (R^2^ = 0.05)			Total Vegetables (R^2^ = 0.07)			Empty Calories (R^2^ = 0.06)		
	b	SE	*p*	b	SE	*p*	b	SE	*p*	b	SE	*p*	b	SE	*p*
Intercept	59.91	7.67	<0.001	2.37	1.06	0.03	2.38	1.05	0.02	3.72	0.85	<0.001	9.08	3.42	0.008
Male	−2.56	1.53	0.10	−0.19	0.21	0.38	−0.30	0.21	0.15	−0.70	0.17	<0.001	−0.87	0.68	0.20
Age, years	0.21	0.09	0.02	0.03	0.01	0.01	0.03	0.01	0.02	0.01	0.01	0.45	0.08	0.04	0.05
Other race (not African-American)	0.93	1.90	0.62	−0.43	0.26	0.10	−0.12	0.26	0.64	0.06	0.21	0.79	1.37	0.85	0.11
College graduate	0.95	1.09	0.39	0.34	0.15	0.03	0.29	0.15	0.06	0.24	0.12	0.05	0.87	0.49	0.07
Married or unmarried couple	−0.56	1.21	0.64	0.11	0.17	0.50	−0.02	0.17	0.91	0.10	0.13	0.45	−0.71	0.54	0.19
Employed full or part-time	3.26	1.56	0.04	0.30	0.22	0.16	0.40	0.21	0.06	0.10	0.17	0.58	1.41	0.69	0.04
BMI, kg/m^2^	−0.20	0.10	0.04	−0.02	0.01	0.10	−0.01	0.01	0.37	0.00	0.01	0.82	−0.06	0.04	0.19
WOMAC global score ^a^	−0.04	0.03	0.16	−0.01	0.00	0.09	−0.01	0.00	0.08	−0.01	0.00	0.06	−0.02	0.01	0.17
	**Greens and Beans (R^2^ = 0.03)**			**Whole Grains (R^2^ = 0.03)**			**Dairy (R^2^ = 0.06)**			**Total Protein Foods (R^2^ = 0.03)**					
	**b**	**SE**	***p***	**b**	**SE**	***p***	**b**	**SE**	***p***	**b**	**SE**	***p***			
Intercept	3.96	0.97	<0.001	3.37	2.13	0.11	4.12	1.69	0.02	4.95	0.47	<0.001			
Male	−0.53	0.19	0.006	−0.15	0.42	0.73	0.08	0.34	0.82	0.22	0.09	0.02			
Age, years	0.01	0.01	0.42	0.02	0.03	0.45	0.00	0.02	0.94	−0.01	0.01	0.11			
Race not African-American	0.03	0.24	0.92	1.17	0.53	0.03	1.49	0.42	<0.001	−0.11	0.12	0.34			
College graduate	0.08	0.14	0.54	−0.76	0.30	0.01	−0.30	0.24	0.21	0.08	0.07	0.24			
Married or unmarried couple	0.24	0.15	0.12	−0.34	0.34	0.31	−0.48	0.27	0.07	0.00	0.07	0.99			
Employed full or part-time	0.08	0.20	0.69	0.52	0.43	0.23	−0.32	0.34	0.35	0.06	0.10	0.50			
BMI, kg/m^2^	−0.01	0.01	0.43	0.00	0.03	0.90	−0.01	0.02	0.58	0.01	0.01	0.16			
WOMAC global score ^a^	0.00	0.00	0.77	0.00	0.01	0.78	0.02	0.01	0.01	0.00	0.00	0.66			
	**Seafood and Plant Proteins (R^2^ = 0.03)**			**Fatty Acids (R^2^ = 0.04)**			**Refined Grains (R^2^ = 0.06)**			**Sodium (R^2^ = 0.02)**					
	**b**	**SE**	***p***	**b**	**SE**	***p***	**b**	**SE**	***p***	**b**	**SE**	***p***			
Intercept	3.46	0.90	<0.001	8.19	1.79	<0.001	9.85	1.14	<0.001	4.45	2.22	0.046			
Male	−0.12	0.18	0.52	−0.36	0.36	0.31	0.00	0.23	0.98	0.37	0.44	0.41			
Age, years	0.02	0.01	0.13	0.01	0.02	0.71	0.00	0.01	0.78	0.01	0.03	0.57			
Race not African-American	0.23	0.22	0.30	−0.70	0.44	0.12	−1.22	0.28	<0.001	−0.82	0.55	0.14			
College graduate	0.23	0.13	0.08	−0.10	0.26	0.69	−0.17	0.16	0.28	0.16	0.32	0.61			
Married or unmarried couple	0.05	0.14	0.75	0.25	0.28	0.39	0.16	0.18	0.37	0.09	0.35	0.80			
Employed full or part-time	0.22	0.18	0.23	0.61	0.36	0.09	0.22	0.23	0.34	−0.34	0.45	0.45			
BMI, kg/m^2^	−0.02	0.01	0.18	−0.02	0.02	0.34	−0.02	0.01	0.09	−0.03	0.03	0.35			
WOMAC global score ^a^	0.00	0.00	0.46	−0.02	0.01	0.02	0.00	0.00	0.36	0.00	0.01	0.61			

From linear regression models with HEI-2010 or components as the dependent variable and the variables shown as independent variables. *n* = 400; records with estimated energy <500 or >5000 were excluded from the analysis (*n* = 13). ^a^ A higher score indicates greater difficulties due to OA. Abbreviations: b, beta; BMI, body mass index; WOMAC, Western Ontario McMaster Universities Osteoarthritis Index.
